# Emergence and Evolution of Novel Canine-Avian Reassortant H3N2 Influenza A Viruses in Duck in Leizhou Peninsula, China

**DOI:** 10.3389/fmicb.2022.857800

**Published:** 2022-04-05

**Authors:** Qiucheng Yao, Wenhong Mai, Yuexiao Lian, Mengdi Zhang, Qiang Yao, Caiyun Huang, Ye Ge, Zhihui Zhao

**Affiliations:** ^1^College of Coastal Agricultural Sciences, Guangdong Ocean University, Zhanjiang, China; ^2^Guangdong Laboratory Animals Monitoring Institute and Guangdong Key Laboratory of Laboratory Animals, Guangzhou, China; ^3^China Animal Disease Prevention and Control Center, Beijing, China; ^4^Central People’s Hospital of Zhanjiang, Zhanjiang, China

**Keywords:** avian influenza virus, H3N2, canine originated, evolution, reassortment

## Abstract

Avian-to-mammal transmission and mammalian adaptation of avian influenza virus (AIV) are threats to public health and of great concern. The H3 subtype of influenza virus has low pathogenicity and is widely distributed in humans, canines, equines and avians. In 2018–2019, we isolated six H3N2 subtype influenza viruses from 329 samples acquired from ducks on the Leizhou Peninsula, China, as part of an ongoing virus surveillance program. All viruses were analyzed by whole-genome sequencing with subsequent genetic comparison and phylogenetic analysis. Phylogenetic analysis demonstrated that reassortment of these viruses has occurred among different hosts and subtypes. Some of the H3 AIV isolates have similar genes as subtypes H5 and H7 of highly pathogenic avian influenza viruses (HPAIVs). Most importantly, one strain of H3N2 virus is a novel reassortant influenza virus containing HA and PB2 segments from canine H3N2 virus. The time of most recent common ancestor (tMRCA) data indicated that this reassortant H3N2 virus might have emerged in 2011–2018. The findings suggest that the viruses studied here have undergone multiple reassortment events. Our results provide a framework for understanding the molecular basis of host-range shifts of influenza viruses and we should pay more attention to canine which lived with avian together.

## Introduction

Avian influenza virus (AIV) is a negative-sense RNA virus that belongs to the Orthomyxoviridae family and includes subtypes with a wide range of hosts, primarily birds and mammals (Zhu et al., 2015; Lycett et al., 2019). AIVs are divided into two categories: lowly pathogenic AIVs (LPAIVs) and highly pathogenic AIVs (HPAIVs) (Sun et al., 2017). HPAIVs are leading threats on commercial poultry farms, causing massive economic losses and posing high risks to public health. HPAIVs often cause severe disease outbreaks in domestic poultry and wild birds and can cause outbreaks in humans. LPAIVs usually cause only asymptomatic infection, where infected animals seem healthy, or mild disease in poultry; however, LPAIVs also pose a threat to human health, as they can provide HPAIVs with gene segments, allowing novel HPAIVs or other viruses to arise through genetic rearrangement and recombination and cause severe infection (Peng et al., 2013; Sun et al., 2017). However, as LPAIVs usually do not cause disease or death in animals, they are low-priority targets for animal disease control, which allows them to evolve silently in nature.

H3N2 influenza viruses are the main causes of epidemics and have spread widely in humans since 1968. The H3N2 subtype of influenza virus is widespread in humans, canines, ferrets, swine, and domestic and wild birds. Although the H3N2 subtype is categorized as an LPAIV, it actively circulates in humans. Some avian-origin H3N2 viruses have been transmitted to dogs, causing severe respiratory disease. In 2007, Song et al. (2008) and Zhang et al. (2015) isolated H3N2 strains of avian origin from dogs with severe respiratory diseases and reported that this subtype of AIV could bind the SAα 2,3-gal receptor (revealing that this subtype can be transmitted from poultry to dogs and steadily persist in dogs). During 2009–2010, 12 avian-origin H3N2 strains were isolated from dogs in northern China. Analysis of eight gene segments of the isolates indicated that all eight segments were from H3N2 AIVs from southern China and Korea (Sun et al., 2013a). These studies show that the H3N2 subtype has spread globally and that dogs play an important role as intermediate hosts.

Virus ecology and evolution play central roles in disease emergence. A critical feature of the ecology and epidemiology of AIVs is interspecies transmission (Webster, 1998). Different lineages of influenza virus have jumped to different hosts or undergone reassortment, sometimes resulting in pandemics. During its circulation among migratory wild birds, H3N2 AIV can donate its gene segments to other viruses. Studies have clearly revealed H3N2 viruses transmitted among guinea pigs and ferrets via respiratory droplets and a severe influenza outbreak occurring as a result in chickens in China in 2017 (Guan et al., 2019). In addition, different lineages of H3N2 influenza viruses have been found in several animals, including horses, birds, pigs and dogs, and they not only have accelerated AIV reassortment and interspecies transmission to humans but also provide potential for other zoonotic infections, posing high risks to the poultry industry and public health (Peng et al., 2013; Parrish et al., 2015; Yang et al., 2015; Zhu et al., 2015; Cui et al., 2016; Li et al., 2016; Sun et al., 2017).

In this study, we investigated the phylogenetic and evolutionary dynamics of H3N2 AIV in the Leizhou Peninsula to accurately identify its origin and genotypes as well as its cross-species transmission and spread in new host populations. Notably, our results enhance our understanding of what has sustained canine and avian transmission of H3N2 influenza virus and the mechanism of emergence of novel influenza viruses in new hosts. Our research provides basic and valuable information for achieving the comprehensive control of AIVs and has great significance for the maintenance of human health.

## Materials and Methods

### Sample Collection

A total of 329 oropharyngeal and cloacal swab samples were collected from apparently healthy ducks on six duck farms on the Leizhou Peninsula, China. In addition, tissue samples were collected from diseased or dead poultry on farms during the study. Each oropharyngeal or cloacal swab was placed into a collection tube containing 1 ml of phosphate-buffered saline (PBS; containing 1,000 U/ml penicillin and 2,000 μg/ml streptomycin). The sample tubes were stored in a handheld portable 4°C refrigerator, transported to the laboratory within 24 h and then immediately frozen at −80°C.

### Virus Isolation and Identification

The samples were inoculated into 9–11-day-old SPF chicken embryos using a virus isolation manual. After 48 h of culture, the allantoic fluid of the surviving chicken embryos was harvested and tested for hemagglutination activity with 1% chicken red blood cells (cRBCs). The viruses were purified three times by serial dilution and inoculated into 9–11 days old SPF chicken embryos. The hemagglutinin (HA) subtype was identified by the hemagglutination inhibition (HI) method and sequencing. Neuraminidase (NA) subtypes were determined by direct sequencing. RNA extraction, RT-PCR, subtyping of extracted RNA (Invitrogen) and reverse transcription of viral RNA (Invitrogen) were performed according to the manufacturers’ instructions. RT-PCR was performed according to the method routine (Shi et al., 2018). The primers used in these studies to identify HA and NA subtypes by RT-PCR would be supported if necessary. Sanger sequencing was used to determine the whole-genome sequences of the AIV isolates. The data were merged and assembled.

### Sequence Information

A total of 100 HA sequences of the H3 subtype of AIV; 138 NA sequences of the N2 subtype of AIV; and 67 H3N2 canine influenza virus (CIV) HA and NA, 10 H3N2 feline influenza virus (FIV) HA and NA, 1 H7N2 FIV HA or 1 H7N2 FIV NA, and 82 H3N8 equine influenza virus (EIV) HA and NA coding regions were collected from the GISAID database^1^ and the NCBI GenBank database^2^.

### Alignment and Model Selection

The length of each segment after alignment was as follows: HA, 1,698 nucleotides (nt); M, 1,044 nt; NA, 1,407 nt; NP, 1,494 nt; NS, 1,051 nt; PA, 2,148 nt; PB1, 2,271 nt; and PB2, 2,277 nt. BLASTn was performed locally with default parameters against the downloaded sequences using each of the H3N2 genome sequences from this study as a query. The first 100 gene sequences in the BLAST output were collected. For each gene, multiple sequence alignment was performed using Muscle and included the H3N2 sequences from this study.

Phylogenetic analysis was performed for two rounds using RAxML. In the first round, 1,000 bootstrap replicates were run, and the best-fit model according to the Bayesian information criterion (BIC) of each gene segment was selected as the nucleotide substitution model (Kalyaanamoorthy et al., 2017). Based on the phylogenetic topologies obtained and their bootstrap values, we selected a few representative reference sequences and formed eight smaller data sets. TempEst (version 1.5.1) was used to analyze the temporal signal in the selected sequences.

### Phylogenetic and Evolutionary Dynamics Analyses

Phylogenetic analysis was repeated using the method described above. For all eight datasets, sequences without full alignment length were removed. A phylogenetic tree was reconstructed by the maximum likelihood (ML) method in RAxML under the GTRGAMMA model with 1,000 bootstrap replicates. The time of the most common ancestor (tMRCA) was estimated with the BEAST package (v1.8.4) with the suitable nucleotide substitution model and clock model and a constant size coalescent model. A Markov chain Monte Carlo (MCMC) run of 50,000,000 states sampling each 5,000 steps was performed to obtain an effective sample size (ESS) of ≥ 200. Each tree was run twice independently and combined using LogCombiner. After a burn-in of 10%, the final tree was summarized using TreeAnnotator and visualized in FigTree. Root-to-tip genetic distance analysis was performed based on the ML tree against sampling dates using TempEst. The maximum clade credibility (MCC) trees of each segment were built using the same methods as above. The tMRCA and evolutionary rates were estimated using the Bayesian MCMC process. The MCC trees were reconstructed with 10% burn-in and annotated with FigTree (v1.4.1).

### Amino Acid Analysis

Amino acid changes associated with canine infection of H3N2 CIV and H3N8 CIV were analyzed using MEGA 7.0. Consensus sequences were aligned, and mutations were recorded. The positions of the mutations of each enzootic cluster were confirmed manually. The number of amino acid changes in each enzootic cluster was counted.

## Results

### Sampling, Virus Isolation, and Sequencing

**FIGURE 1:**
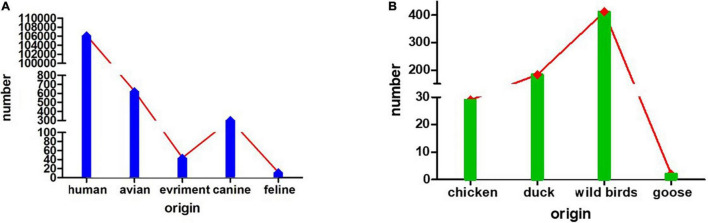
The numbers of isolated H3N2 viruses. **(A)** The numbers of H3N2 viruses isolated from humans, avians, the environment, canines and felines. **(B)** The numbers of H3N2 viruses isolated from avians, such as chickens, ducks, wild birds, and geese.

Based on HA testing and RT-PCR, in total, six H3N2 subtype AIVs were isolated from samples collected from ducks on the Leizhou Peninsula, Guangdong Province, China, from 2018 to 2019. One strain was isolated in 2018, and the other five strains were isolated in 2019. The exact isolation time, place and host of each isolate are listed in Table 1. We analyzed the complete genome sequences of these H3N2 subtype AIVs and submitted them to the GenBank database. To date, the H3N2 subtype of AIV has been detected in many species, such as humans, avians, canines, and felines, and in the environment. Avians are the second largest reservoir of H3N2, with chickens, ducks, geese, and wild birds being the main hosts. Among them, wild birds represent more than half of H3N2 hosts (Figures 1A,B).

**TABLE 1:** Information of the 6 avian influenza viruses.

No.	Virus name	Abbreviation	Time	Place	Host
1	A/duck/Guangdong/A1-2/2019 (H3N2)	A1-2/H3N2	2018.10	Wuling duck farm, Suixi, Zhanjiang, Guangdong Province	Duck
2	A/duck/Guangdong/SN3/2019 (H3N2)	SN3/H3N2	2019.03	Shannei duck farm, Suixi, Zhanjiang, Guangdong Province	Duck
3	A/duck/Guangdong/PY8/2019 (H3N2)	PY8/H3N2	2019.10	Puyang duck farm, Leizhou, Zhanjiang, Guangdong Province	Duck
4	A/duck/Guangdong/XY3/2019 (H3N2)	XY4/H3N2	2019.10	Xiayang duck farm, Leizhou, Zhanjiang, Guangdong Province	Duck
5	A/duck/Guangdong/XY44/2019 (H3N2)	XY44/H3N2	2019.10	Xiayang duck farm, Leizhou, Zhanjiang, Guangdong Province	Duck
6	A/duck/Guangdong/XY46/2019 (H3N2)	XY46/H3N2	2019.10	Xiayang duck farm, Leizhou, Zhanjiang, Guangdong Province	Duck

### Phylogenetic Analysis of H3N2 Avian Influenza Virus

Phylogenetic analysis of the eight AIV genes in this study was performed to assess their relationships with corresponding genes in domestic poultry, wild birds and other animals using data from both the NCBI and GISAID databases. The criterion for grouping the viruses was the identity above 95%, which were belong to one group. The results revealed that the eight genes (PB2, PB1, PA, HA, NP, NA, M, and NS) were distributed in Eurasian lineages.

**FIGURE 2:**
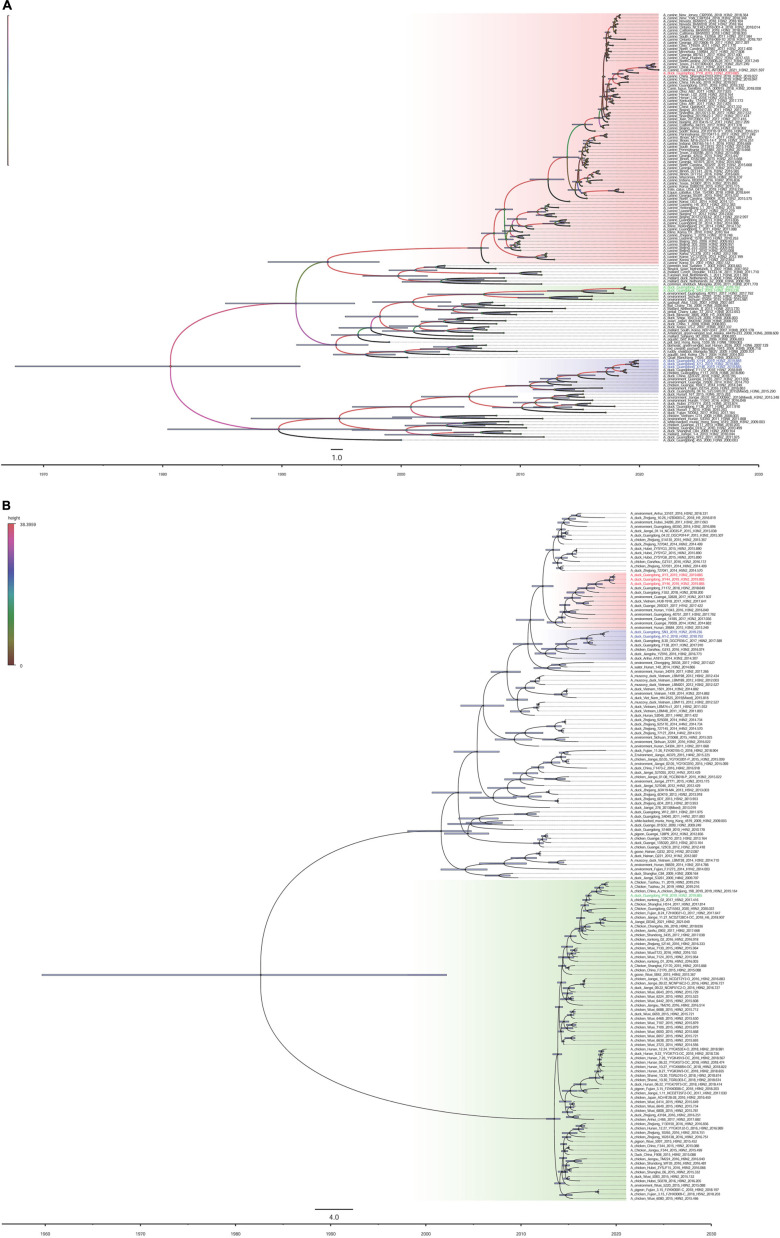
Maximum clade credibility trees of the coding sequences of 2 segments. Node bars indicate the 95% highest posterior density (HPD) of node height. The influenza A virus strains isolated in this study are colored red, blue, and green. Each branch was colored by posterior probability. The segments shown are **(A)** hemagglutinin (HA); **(B)** neuraminidase (NA).

**FIGURE 3:**
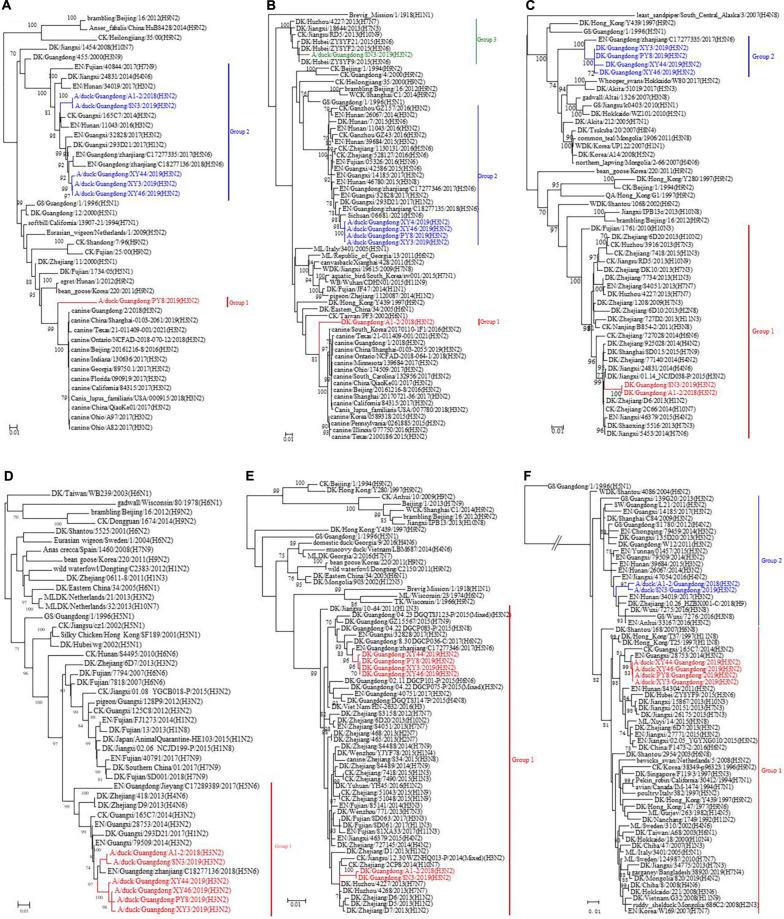
Phylogenetic analysis of the surface genes of H3N2-subtype AIVs isolated from 2018 to 2019 using the maximum likelihood method. **(A)** PB2, **(B)** PB1, **(C)** PA, **(D)** NP, **(E)** M, and **(F)** NS. Phylogenetic trees were generated with the MAGE 7.0 software package. The evolutionary history was inferred using the maximum likelihood method based on the Tamura-Nei model. The tree with the highest log likelihood (–10541.1792) is shown. The percentage of trees in which the associated taxa clustered together is shown next to the branches. The initial tree(s) for the heuristic search were obtained by applying the neighbor-joining method to a matrix of pairwise distances estimated using the maximum composite likelihood (MCL) approach. The tree is drawn to scale, and the branch lengths indicate the number of substitutions per site. All positions containing gaps and missing data were eliminated. The phylogenetic trees of the 6 genes were obtained without a root tree. The sequences of viruses listed in black were downloaded from available databases. The viruses listed in red, blue, and green were sequenced in this study. CK, chicken; DK, duck; GS, goose; SW, swine; ML, mallard; WDK, wild duck; EN, environment.

The surface genes (HA and NA) of the viruses we named XY3/H3N2, XY44/H3N2, and XY46/H3N2 exhibited the highest sequence homologies with A/duck/Guangdong/F352/2018 (H3N2) and A/duck/Guangdong/F1172/2018 (H3N2). The HA and NA sequences of the PY8/H3N2 virus exhibited the highest homology with A/canine/Guangdong/3/2018 (H3N2) and A/chicken/nantong/02/2017 (H9N2), respectively (Figures 2A,B). However, the inner genes (PB1, PA, M, NS) of the strains PY8/H3N2, XY3/H3N2, XY44/H3N2, and XY46/H3N2 exhibited the highest sequence homology with A/Environment/Guangdong/zhanjiang/C18277136 (H5N6), A/Environment/Guangdong/zhanjiang/C18277135/2018 (H5N6), A/Environment/Guangdong/zhanjiang/C17277346/2017 (H5N6), and A/duck/Guangxi/04.10JX013/2015 (Mixed) (H6N2), respectively (Figures 3B,C,E,F). For the PB2 gene sequence, XY3/H3N2, XY44/H3N2, and XY46/H3N2 exhibited the highest homology with A/Environment/Guangdong/zhanjiang/C17277335/2017 (H5N6), whereas PY8/H3N2 exhibited the highest homology with A/canine/Beijing/20170512-122/2017 (H3N2) (Figure 3A). For the NP gene, PY8/H3N2, XY3/H3N2, and XY44/H3N2 exhibited the highest homology with A/Environment/Guangdong/zhanjiang/C17277346/2017 (H5N6), whereas XY46/H3N2 exhibited the highest homology with A/environment/Guangxi/79509/2014 (H3N2) (Figure 3D). In conclusion, XY3/H3N2, XY44/H3N2, XY46/H3N2 and PY8/H3N2 were closely related to H5-subtype AIVs.

For the A1-2/H3N2 and SN3/H3N2 strains, we analyzed the sequences of the eight gene segments and found that the surface genes (HA and NA) of these two viruses had the highest homology with A/Environment/Hunan/46780/2015 (H3N8) and A/duck/Guangdong/F138/2017 (H3N2), respectively. However, the inner genes (PA, NP, and M) exhibited the highest homology with A/duck/Jiangxi/01.14NCJD040-P/2015 (Mixed) (H1N3), A/Environment/Guangdong/zhanjiang/C17277335/2017 (H5N6), and A/duck/Huzhou/4227/2013 (H7N7), respectively. With respect to the sequences of the inner genes PB2, PB1, and NS, the novel isolated virus A1-2/H3N2 had the highest homology with A/Envrionment/Guangdong/zhanjiang/C17277346/2017 (H5N6), A/canine/Guangdong/1/2018 (H3N2), and A/chicken/Ganzhou/GZ43/2016 (H3N2), respectively, whereas SN3/H3N2 had the highest homology with A/Environment/Guangxi/28753/2014 (H3N2), A/duck/Hubei/ZYSYF2/2015 (H3N6) and A/Environment/Guangxi/79509/2014 (H3N2), respectively. We noticed that both viruses showed close relationships with the H1, H5, and H7 subtypes of AIVs. A Bayesian MCMC method was then utilized to estimate the evolutionary rate for each gene segment (Table 2).

**TABLE 2:** Evolution rate and time of most recent common ancestor for each of the eight segments.

Segment	Correlation coefficient	Best-fit model	Strains	Mean evolutionrate (substitution/site/year)	95% HPD interval	Most recent common ancestor	tMRCA§	95% HPD interval	Posterior probability
PB2	0.9654	GTR+F+I+G4	PY8	2.74E-03	2.1746E-3, 3.331E-3	Beijing canine H3N2 strain	August 2011	(January 2007, September 2015)	0.9827
			XY3			Zhanjiang environment H5N6 strain	October 2018	(July 2015, May 2017)	0.9973
			XY44						
			XY46						
			A1-2			Zhanjiang environment H5N6 strain	July 2018	(January 2018, October 2018)	0.4138
			SN3			Guangxi environment H3N2 strain			
PB1	0.9592	GTR+F+G4	PY8	3.36E-03	2.59E-3, 4.1063E-3	Zhanjiang environment H5N6 strain	September 2016	(January 2016, May 2017)	1
			XY3						
			XY44						
			XY46						
			A1-2			Guangdong canine H3N2 strain	September 2005	(April 2000, January 2011)	
			SN3			Hubei duck H3N6 strain	January 2014	(October 2013, March 2014)	
PA	0.8372	GTR+F+G4	PY8	2.22E-03	1.976E-3, 2.4213E-3	Zhanjiang environment H5N6 strain	June 2013	(March 2012, August 2014)	1
			XY3						
			XY44						
			XY46						
			A1-2			Jiangxi duck H1N3 strain	August 2013	(January 2013, April 2014)	0.866
			SN3						
HA	0.8361	TIM+F+G4	PY8	2.958E-03	2.4021E-3, 3.5226E-3	Guangdong canine H3N2 strain	November 2018	(April 2018, June 2019)	0.9994
			XY3			Guangdong duck H3N2 strain	October 2017	(February 2017, May 2018)	0.9398
			XY44						
			XY46						
			A1-2			Hunan environment H3N8 strain	March 2015	(April 2012, April 2017)	1
			SN3						
NP	0.9567	TVM+F+G4	PY8	4.55E-03	3.6033E-3, 5.5667E-3	Zhanjiang environment H5N6 strain	January 2016	(January 2015, December 2016)	1
			XY3						
			XY44						
			XY46			Guangxi environment H3N2 strain			
			A1-2			Zhanjiang environment H5N6 strain	December 2016	(March 2016, August 2017)	0.996
			SN3						
N2	0.9716	GTR+F+I+G4	PY8	3.58E-03	2.9104E-3, 4.2725E-3	Nantong chicken H9N2 strain	December 2017	(May 2017, June 2018)	1
			XY3			Guangdong duck H3N2 strain	November 2017	(December 2016, July 2018)	
			XY44						
			XY46						
			A1-2				December 2016	(April 2016, June 2017)	
			SN3						
M	0.8319	SYM+G4	PY8	3.32E-03	2.1996E-3, 4.5981E-3	Zhanjiang environment H5N6 strain	December 2016	(June 2016, June 2017)	1
			XY3				November 2016	(March 2016, July 2017)	0.9997
			XY44						
			XY46						
			A1-2			Huzhou duck H7N7 strain	February 2016	(July 2015, July 2016)	0.3266
			SN3						
NS	0.7598	TN+F+G4	PY8	1.86E-03	1.463E-3, 2.2967E-3	Guangxi duck H6N2 strain	June 2014	(June 2013, March 2015)	0.9975
			XY3						
			XY44						
			XY46						
			A1-2			Ganzhou chicken H3N2 strain	May 2013	(February 2012, August 2014)	0.4275
			SN3			Guangxi environment H3N2 strain			

*PB2, basic polymerase 2; PB1, basic polymerase 1; PA, acidic polymerase; HA, hemagglutinin; NP, nucleoprotein; NA, neuraminidase; M, matrix protein; NS, non-structural protein. HPD, highest posterior density. tMRCA, time of most recent common ancestor.*

### Insights Into the Origin of H3N2 Avian Influenza Virus

To determine the origins of the six isolated H3N2 viruses, we sequenced the six inner gene and constructed individual ML phylogenetic trees (Figures 3A–F). The PB1, PA, NP, M, and NS gene segments of PY8/H3N2 were closely related to those of an H5N6 strain isolated from the environment in Zhanjiang, China (Figures 3B–F). The HA and PB2 gene sequences were closely related to sequences of strains isolated from canines in Beijing and Guangdong, respectively, in China (Figures 2A, 3A). The NA gene sequences were closely related to sequences from an H9N2 strain isolated from chicken in Nantong, China (Figure 2B). The PB2, PB1, PA, NP, and M sequences of XY3/H3N2, XY44/H3N2 and XY46/H3N2 were all closely related to those of an H5N6 strain isolated from the environment in Zhanjiang, China. The HA and NA sequences were closely related to those of H3N2 and H6N2 strains, respectively, isolated from Guangdong ducks. The PB1 gene segments of A1-2/H3N2 and SN3/H3N2 were closely related to those of duck H3N6 isolated in Hubei Province, China. The PB2 gene segments of A1-2/H3N2 and SN3/H3N2 were closely related to those of environment-isolated strains of H5N6 from Zhanjiang and H3N2 from Guangxi, respectively. The PA gene segments A1-2/H3N2 and SN3/H3N2 were closely related to a sequence of duck H1N3 from Jiangxi Province, China.

The NP, M and HA gene segments of A1-2/H3N2 and SN3/H3N2 were closely related to sequences of an H5N6 strain isolated from the environment in Zhanjiang, a H7N7 strain from duck in Huzhou and an H3N8 strain isolated from the environment from Hunan, China, respectively. The N2 gene segments of A1-2/H3N2 and SN3/H3N2 were closely related to a sequence of an H3N2 strain isolated from duck in Guangdong, as were those of XY3/H3N2, XY44/H3N2 and XY46/H3N2. The NS gene segments of A1-2/H3N2 and SN3/H3N2 were closely related to those of an H3N2 strain from Ganzhou chicken and an H3N2 strain from the environment in Guangxi, China (Table 1). These results showed that the six H3N2 viruses were all reassortant strains carrying gene segments from various influenza A virus subtypes and various hosts, including avians and mammals (canines).

BEAST software was used to determine the tMRCA. Reassortment events to produce the novel H3N2 viruses occurred between 2007 and 2019 (Figures 2A,B). The time span was long, especially for the PY8/H3N2 virus. Most of the gene sequences arose in 2017–2019; however, some of them emerged earlier. The PB1 sequence of the A1-2/H3N2 strain emerged in June 2007, and the PB2 sequence of the PY8/H3N2 strain emerged in August 2011 (Table 2). The correlation coefficient of HA and NA were 0.8361 and 0.9716, respectively. The correlation coefficient of the other genes were all above 0.7 (Table 2). In addition, using BEAST software to determine the tMRCA, we found that the PB2, PB1, NP and M segments of the H5N6 viruses isolated from the environment in Zhanjiang most likely emerged in April-July 2017 and that the PA gene segments emerged in June 2013. These findings indicated that these H3N2 genes were possibly in circulation in the Zhanjiang poultry AIV gene pool at the beginning of 2013. Furthermore, these findings indicated that the novel waterfowl-origin H3N2 viruses are reassortant influenza viruses containing segments from a multitude of subtypes and different host species (various wild waterfowl, domestic poultry). In this study, the mean substitution rate of the H3N2 HA gene was 2.42 × 10^–3^ (1.7195–3.1847 × 10^–3^, 95% HPD) substitutions/site/year, and among the eight genes of H3N2 AIV, the HA gene was characterized by a slow evolution rate. The fastest rate was detected for the NP gene, at 4.55 × 10^–3^ (3.6033–5.5667 × 10^–3^, 95% HPD) substitutions/site/year (Table 1).

### Molecular Characterization of Surface Genes

We analyzed the genome sequences of the surface genes of the six isolated viruses to determine whether the viruses had acquired genetic markers associated with mammalian pathogenicity, virulence, or adaptation to new hosts. None of the six isolated viruses had consecutive basic amino acids in their sequences. The HA protein sequences belonging to A1-2/H3N2, SN3/H3N2, XY3/H3N2, XY44/H3N2, and XY46/H3N2 shared the cleavage site are PEKQTR↓GLF; that of PY8/H3N2 had a different cleavage site (PERQTR↓GLF). The cleavage site of PY8/H3N2 is the same as that of a CIV isolated from Jiangsu, China (Lin et al., 2012). However, the protein sequences of all the strains corresponded to low-pathogenicity AIV. The amino acid substitutions Q226L and G228S (H3 numbering, which is used throughout the manuscript) are the key substitutions that play an important role in recognizing the receptors of humans (Ge et al., 2018; Guan et al., 2019; Shi et al., 2020). The substitution of A138S in the HA protein could contribute to the virulence of influenza virus to mammalian hosts (Le et al., 2019). In this study, no substitutions of A138S, Q226L, or G228S were detected in any of the six isolated viruses. Some researchers demonstrated that the amino acid substitution 155T in the HA protein altered the binding of the human-type receptor in the H9N2 virus and that the H9N2 virus was the donor of inner gene segments to the H7N9 and H10N8 viruses, which infected humans in China (Li et al., 2014; Guan et al., 2019). We compared the protein sequences of the six isolated viruses and found that all of them exhibited the 155T substitution, which indicated that these H3N2 AIVs may have an increased ability to bind the human-type receptor.

Some studies proved that the deletion of amino acids at positions 63–65 confers the virus enhanced lethality against mice (Sun et al., 2013b; Shi et al., 2020). All six isolated strains except PY8/H3N2 lacked deletions in the NA protein sequence. Some substitutions in the NA protein sequence, including V116A, E119G, Q136L, R152K, H274Y, and R292K, have been shown to confer AIV acquired resistance against neuraminidase inhibitors (Boltz et al., 2010; Hurt et al., 2010; Gubareva et al., 2017; Kode et al., 2019a; Shi et al., 2020). We found none of the above mutations in the NA active sites. Kode et al. (2019b) found the novel I117T substitution in HPAIV and showed that it conferred reduced susceptibility to oseltamivir and zanamivir. Interestingly, we also found this mutation in our six isolated viruses (Table 3).

**TABLE 3:** Molecular characterization of six isolates presented in this study.

Viral protein	A1-2	SN3	PY8	XY3	XY44	XY46	Comments[reference]
HA(H3 numbering)	PEKQTR↓GLF	PEKQTR↓GLF	PERQTR↓GLF	PEKQTR↓GLF	PEKQTR↓GLF	PEKQTR↓GLF	Multi-basic cleavage site
	138A^※^	138A	138A	138A	138A	138A	A→S: contributes virulence from influenza virus to mammalian hosts (Le et al., 2019)
	155T	155T	155T	155T	155T	155T	I→T: increases affinity for the human-type receptor (Li et al., 2014; Guan et al., 2019)
	226Q	226Q	226Q	226Q	226Q	226Q	Q→L: increases binding to human-type influenza receptor (Ge et al., 2018; Guan et al., 2019; Shi et al., 2020)
	228G	228G	228G	228G	228G	228G	G→S: increases binding to human-type influenza receptor (Ge et al., 2018; Guan et al., 2019; Shi et al., 2020)
NA	–	–	Amino acid necklace deletion	–	–	–	63–65: Enhances virus lethality in mice (Sun et al., 2013b; Shi et al., 2020)
	116V	116V	116V	116V	116V	116V	V→A: Resistance to neuraminidase inhibitors (Boltz et al., 2010)
	117T	117T	117T	117T	117T	117T	I→T: Reduces susceptibility to oseltamivir and zanamivir (Kode et al., 2019b)
	119E	119E	119E	119E	119E	119E	E→G: resistance to neuraminidase inhibitors (Shi et al., 2020)
	136Q	136Q	136Q	136Q	136Q	136Q	Q→L: Resistance to neuraminidase inhibitors (Hurt et al., 2010)
	152R	152R	152R	152R	152R	152R	R→K: Resistance to neuraminidase inhibitors (Gubareva et al., 2017; Shi et al., 2020)
	274H	274H	274H	274H	274H	274H	H→Y: Resistance to neuraminidase inhibitors (Gubareva et al., 2017; Shi et al., 2020)
	292R	292R	292R	292R	292R	292R	R→K: Resistance to neuraminidase inhibitors (Gubareva et al., 2017; Shi et al., 2020)
PB2	89V	89V	89V	89V	89V	89V	L→V: Adaptation to mammalian host (Ge et al., 2018)
	627E	627E	627E	627E	627E	627E	E→K: Adaptation to mammalian host (Ge et al., 2018)
	701D	701D	701D	701D	701D	701D	D→N: Increases virulence and host range (Yang et al., 2021)
PB1	622G	622G	622G	622G	622G	622G	D→G: Increases polymerase activity and virulence in mice (Feng et al., 2016)
PB1-F2	66N	66N	66N	66N	66N	66N	N→S: Increases replication, virulence and antiviral response in mice (Ge et al., 2017; Suttie et al., 2019; Pawestri et al., 2020)
PA	97T	97T	97T	97T	97T	97T	T→I: Enhances polymerase and virulence (Yang et al., 2019)
PA-X	195K	195K	195K	195K	195K	195K	R?K: Enhances the virulence in mammals (Sun et al., 2020; Trinh et al., 2020)
NP	319N	319N	319N	319N	319N	319N	N→K: Increases virulence in mice and mammalian cells (Na et al., 2021)
M1	30D	30D	30D	30D	30D	30D	N→D: Increases virulence in mice and mammalian cells (Li et al., 2014; Zhang et al., 2019)
	215A	215A	215A	215A	215A	215A	T→A:Increases virulence in mice and mammalian cells (Zhang et al., 2019; Na et al., 2021)
M2	31S	31S	31N	31N	31S	31N	S→N: Amantadine resistance (Mei et al., 2019; Tuong et al., 2020; Zhang et al., 2021)
NS1	42S	42S	42S	42S	42S	42S	P/A→S: Increases virulence in mammals (Tuong et al., 2020)
NEP/NS2	31M	31M	31M	31M	31M	31M	M→I: Increases virulence in mammals (Trinh et al., 2020)

*^※^Amino acid position. A, alanine; D, aspartic acid; E, glutamic acid; G, glycine; H, histidine; I, isoleucine; K, lysine; L, leucine; M, methionine; N, asparagine; P, proline; Q, glutamine; R, arginine; S, serine; T, threonine; V, valine; S, serine; Y, tyrosine.*

### Molecular Characterization of Inner Genes

The six viruses identified here also possessed the amino acid substitution 251R in polymerase basic 2 (PB2), which has frequently been identified in human influenza viruses. Of note, 251R and 590S in PB2 are known determinants of adaptation to growth in mammals. In the PB2 of CIV H3N2, 2006–2015, the main amino acid at position 251 was K; in the PB2 of CIV H3N2, 2016–2017, it was R. The frequency of 251R in PB2 in influenza virus A (H3N2) in humans and influenza A (H1N1) pdm09 virus is approximately 99%. 590S in PB2 is a known determinant of adaptation to growth in mammals. 590G in PB2 has been identified in CIV H3N2, 2006–2015, whereas 590S has been identified in CIV H3N2, 2016–2017; influenza virus A (H3N2) in humans and influenza A (H1N1) pdm09 virus. The six H3N2 viruses identified in this study had the 590G substitution in the PB2 protein (Lyu et al., 2019). Several substitutions in the PB2 protein of AIV, including L89V, E627K and D701N, have been demonstrated to increase virulence and host range, especially the L89V and E627K substitutions, which confer adaptation to mammalian hosts (Ge et al., 2018; Yang et al., 2021). In this study, the six viruses we isolated had the 89V mutation but not the 627K and 701N mutations in the PB2 protein. The PB2 L89V mutation, which is known to confer adaptation to mammalian hosts, was detected in all six strains.

To determine whether our six H3N2 viruses had the 563R mutation, which is associated with increased virulence and modulation of the host-antiviral IFN response, we analyzed the polymerase basic 1 (PB1) protein. We did not detect the substitution at position 563. The 622G mutation that emerged in PB1 is known to increase polymerase activity and virulence in mice (Feng et al., 2016). We found that all of the six H3N2 strains we isolated showed this mutation in the PB1 protein. The N66S mutation in PB1-F2 is associated with increased replication, virulence, and antiviral response in mice (Ge et al., 2017; Suttie et al., 2019; Pawestri et al., 2020). The six H3N2 stains identified in this study encoded the full-length PB1-F2 protein, but we did not find the 66S mutation in any of the strains.

The T97I substitution in the PA protein has been proven to enhance polymerase activity and virulence (Yang et al., 2019); however, we did not detect the 97I mutation in any of the six H3N2 strains. H9N2 AIV has been the dominant AIV and the dominant epidemic AIV in China for a long time, and the 195K mutation in PA-X amino acids has been proven to enhance the virulence of H9N2 AIV in mammals in previous studies (Sun et al., 2020; Trinh et al., 2020). All the identified strains in this study have this substitution.

Analysis of NP protein from the six H3N2 isolated strains revealed no presence of the 319K mutation, which has been reported to increase virulence to mice and mammalian cells (Na et al., 2021). In addition to having several mutations, including N30D and T215A in the M1 protein, which are known to increase virulence in mice and mammalian cells (Zhang et al., 2019; Na et al., 2021), we found that all six H3N2 strains have D (aspartic acid) and A (alanine) at positions 30 and 215, respectively. Notably, PY8/H3N2, XY3/H3N2, and XY46/H3N2 have the 31N mutation in the M2 protein, which indicates that these strains have amantadine resistance (Mei et al., 2019; Tuong et al., 2020; Zhang et al., 2021). Notably, all six H3N2 viruses have a P/A42S mutation in the NS1 protein, which is associated with increased virulence in mammalian hosts (Tuong et al., 2020). The M31I substitution in the NS2 protein is thought to be associated with increased virulence in mammals (Trinh et al., 2020). However, our isolates did not contain this mutation, which indicated that these strains might have low virulence in mammals.

## Discussion

The natural reservoir of influenza A virus is waterfowl. Normally, waterfowl viruses are not adapted to infect and spread in the human population. Sometimes, through reassortment or whole host-shift events, genetic material from waterfowl viruses is introduced into the human population and causes a worldwide pandemic. AIV poses a threat to both humans and animals. Humans were infected by the H5N1 and H9N2 subtypes of AIV in Southeast Asia in 1997 and the H5N6 subtype of AIV in China, revealing that AIVs can traverse the species barrier from birds to humans (Zou et al., 2020).

The H3N2 virus in chickens from live poultry markets (LPMs) was first detected in Central China in 2001 (Liu et al., 2003). Since 2009, H3N2 AIVs have been regularly reported in China (Zhou et al., 2011). As of September 12, 2021, there were 104255 H3N2 influenza virus strains in the GISAID database, of which only 5,546, including 154 avian-originated strains, were isolated from animals. H3N2 AIVs are LPAIVs, and infectious poultry generally have mild symptoms and lack control and prevention. However, H3N2 AIVs can donate their gene segments to HPAIVs and thereby cause accelerated recombination or mutation, potentially causing the next pandemic and threatening public health. Here, we extensively characterized 6 avian H3N2 viruses that were isolated from duck farms on the Leizhou Peninsula, China, and found that the H3N2 viruses circulating in avian species in nature have undergone frequent reassortment and formed complicated genotypes. Viral reassortment is the source of emergence of deadly HPAI viruses that are transmissible to mammals and are prone to adaptive evolution in their new hosts (Guo et al., 2019). It is still disputed whether the infamous 1918 Spanish flu pandemic was caused by a reassortant strain evolved in mammals or an entirely avian-like virus that adapted to humans (Taubenberger et al., 2005).

**FIGURE 4:**
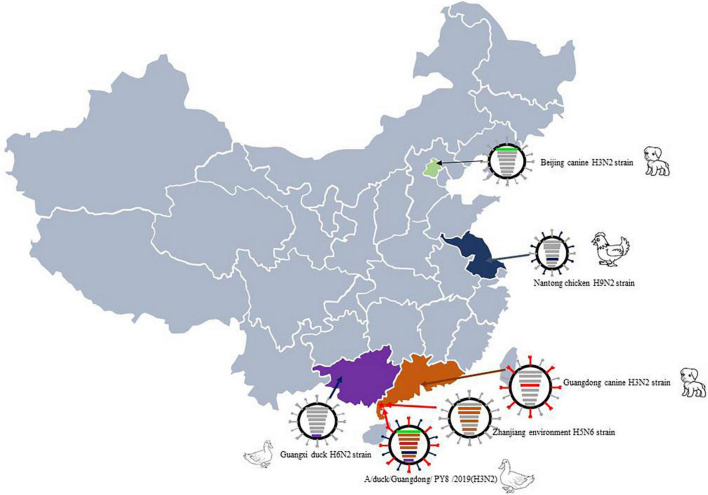
Sampling point and the proposed reassortment and transmission of H3N2. The sampling point is marked with a red star, and the location of the case of canine infection with influenza A subtype H3N2 virus is marked in yellow. Guangdong-origin segments are colored in red, Guangxi-origin segments are colored in purple, and Jiangsu-origin segments are colored in blue.

Exchange of gene segments through reassortment is a major feature of influenza A virus evolution and frequently contributes to the emergence of novel epidemic, pandemic, and zoonotic strains. In particular, some gene segments, such as the HA segment of PY8/H3N2, include surface genes from canines. Therefore, it has been reported that H3N2 CIV originated from avians. Our analyses suggested that the genes can circulate among different hosts and be transmitted from avians to mammals and vice versa. In addition, the tMRCA data indicated that the reassortment leading to the emergence of this H3N2 virus might have occurred in 2017. Importantly, the PB1, PA, NP and M genes of the novel PY8/H3N2 isolate show the highest nucleotide similarities to those of the avian H5N6 strains. The PB2 gene originated from canine virus isolated in Beijing, and the NA and NS genes originated from avian in Jiangsu and Guangxi. These findings suggest that reassortment occurred in wild birds and/or domestic poultry (Figure 4).

The findings suggest a threat of these viruses with respect to influenza virus-acquired ability of transmission across species. During routine monitoring, we found that duck farms were located in remote locations far from residential areas, with little chance of outside contact. However, every duck farm had one or more dogs as guard dogs. We speculate that the viruses with canine-originated gene segments emerged because of long-term close contact between ducks and guard dogs. Additionally, whether dogs, as the closest companions of humans, could facilitate the transmission of CIV to humans needs further investigation. However, we found evidence that canines may have donated canine-origin gene segments to avian influenza viruses that lead to gene reassortment.

Some of the H3 AIV isolates had gene segments (PB2 and N2 genes) similar to those of not only LPAIVs (H3 and H9 AIVs) but also H5 and H7 HPAIVs. This result is consistent with reports that genetic reassortment involving H3 AIV and H5N6 HPAIV appeared in poultry in China. The origin analysis of the six H3N2 viruses isolated in this study indicated that most of the gene segments originated from different subtypes of influenza viruses previously detected in chickens and ducks, suggesting that different influenza viruses circulate together and that gene reassortment occurs frequently among these avian species.

The analysis of the protein sequences of surface and inner genes of the six H3N2 AIVs in this study suggest that circulating AIVs may have the potential to bind the human-type receptor and that antiviral drugs and amantadine may be ineffective for treating poultry infected with these AIVs. All six viruses have the ability to adapt to mammalian hosts and enhance virulence, thus posing severe threats to both public health and poultry markets.

## Conclusion

In conclusion, the analysis of the six strains of AIVs we isolated proved that their genomes have undergone reassortment with the H1N3, H3N8, H5N6, H6N2, H7N7, and H9N2 subtypes of AIV. Our study has thus revealed the risks to human health posed by H3N2 avian viruses and emphasizes the importance of continuous monitoring and evaluation of H3N2 influenza viruses circulating in poultry.

## Data Availability Statement

The datasets presented in this study can be found in online repositories. The names of the repository/repositories and accession number(s) can be found in the article/supplementary material.

## Author Contributions

YG and ZZ were supported the fund. YG performed the data analysis. QuY and WM did the experiment. YL did the evolution analysis. MZ did the part the experiment. QaY revised the manuscript. CH supported the sampling. All authors contributed to the article and approved the submitted version.

## Conflict of Interest

The authors declare that the research was conducted in the absence of any commercial or financial relationships that could be construed as a potential conflict of interest.

## Publisher’s Note

All claims expressed in this article are solely those of the authors and do not necessarily represent those of their affiliated organizations, or those of the publisher, the editors and the reviewers. Any product that may be evaluated in this article, or claim that may be made by its manufacturer, is not guaranteed or endorsed by the publisher.

## Footnotes

1
https://www.gisaid.org

2
https://www.ncbi.nlm.nih.gov/

## Abbreviations

AIV: avian influenza virus
HA: hemagglutinin gene
NA: neuraminidase gene
PB2: polymerase PB2 gene
PB1: polymerase PB1 and putative PB1-F2 protein genes
PA: polymerase PA and PA-X protein (PA-X) genes
M: matrix protein 2 and matrix protein 1 genes
NP: nucleocapsid protein gene
NS: nuclear export protein (NEP) and non-structural protein 1 (NS1) genes.
